# ‘More than devastating’—patient experiences and neurological sequelae of Japanese encephalitis^§^

**DOI:** 10.1093/jtm/taz064

**Published:** 2019-08-28

**Authors:** Lance Turtle, Ava Easton, Sylviane Defres, Mark Ellul, Begona Bovill, Jim Hoyle, Agam Jung, Penny Lewthwaite, Tom Solomon

**Affiliations:** 1 Institute of Infection and Global Health, University of Liverpool, 8 West Derby Street, Liverpool, L69 7BE, UK; 2 NIHR Health Protection Research Unit for Emerging and Zoonotic Infections, University of Liverpool, 8 West Derby Street, Liverpool, L69 7BE, UK; 3 Tropical & Infectious Disease Unit, Royal Liverpool University Hospital, Liverpool, L7 8XP, UK; 4 Encephalitis Society, Malton, North Yorkshire, YO17 7DT, UK; 5 Walton Centre NHS Foundation Trust, Liverpool, L9 7LJ, UK; 6 Tropical and Infectious Diseases, North Bristol NHS Trust, Bristol, Southmead Road, Westbury-on-Trym, BS10 5NB, UK; 7 Neuro-Intensive Care Unit, Royal Hallamshire Hospital, Sheffield, Glossop Rd, S10 2JF, UK; 8 Leeds General Infirmary, Leeds, LS1 3EX, UK; 9 St James’s University Hospital, Leeds Teaching Hospitals NHS Trust, Leeds, Beckett Street, LS9 7TF, UK

**Keywords:** Japanese encephalitis, JE, JE virus, JE vaccine, Japanese encephalitis vaccine, Travel medicine, Traveller

## Abstract

**Background:**

Japanese encephalitis (JE), caused by the mosquito-borne JE virus, is a vaccine-preventable disease endemic to much of Asia. Travellers from non-endemic areas are susceptible if they travel to a JE endemic area. Although the risk to travellers of JE is low, the consequences may be severe.

**Methods:**

Here, we describe three cases of JE in British travellers occurring in 2014–15. In addition, we report, through interviews with survivors and their families, personal experiences of life after JE.

**Results:**

Three cases of JE were diagnosed in British travellers in 2014/15. One was acquired in Thailand, one in China and one in either Thailand, Laos or Cambodia. All three patients suffered severe, life-threatening illnesses, all were admitted to intensive care units and required medical evacuation back to the UK. One patient suffered a cardiac arrest during the acute stage but made a good recovery. The other two patients remain significantly paralysed and ventilator dependent. All three cases had clear indications for vaccination, and all have been left with life-changing neurological sequelae.

**Conclusions:**

Travel health providers should be aware of the severity of JE, as well as the risk, allowing travellers to make fully informed decisions on JE vaccination.

## Introduction

Japanese encephalitis (JE), inflammation and swelling of the brain caused by mosquito-transmitted JE virus (JEV), is the most commonly diagnosed epidemic encephalitis in Asia, predominantly affecting children living in rural areas. Most of South and Southeast Asia is endemic for JE, though local disease incidence can be highly variable. In endemic areas, where exposure usually occurs in childhood, only 0.1–0.3% of infections result in disease,^1^ but in people exposed at an older age, the rate of symptomatic disease may be higher, up to 4% of infections.^2^^,^^3^ The adult population in JE endemic areas is usually immune to JE through asymptomatic exposure to the virus (or vaccination) in childhood,^4^ whereas non-JE immune adults, such as travellers, are susceptible to the disease upon first exposure to JEV, irrespective of age.

JE is a vaccine-preventable disease, and vaccines against JE have been in existence since the 1950s.^5^ Multiple vaccines are currently available, and the World Health Organisation (WHO), US Centers for Disease Control and Prevention (CDC) and Public Health England (PHE) all recommend that JE vaccine should be used in people taking up residence or travelling long term in JE endemic areas, or visiting during the transmission season, if the risk of exposure is deemed to be high, or there is reason to believe the traveller might be predisposed to JE.^6–8^

The improved safety profile of JE vaccines available to travellers^9^^,^^10^ has led to calls for them to be more widely used.^11^ However, JE is rare in travellers, leading to criticism of adopting wider JE vaccine use.^12^ Here, we report three cases occurring in 2014–15, with very severe illness and profound life-changing after-effects. We include narratives from patients and/or their family enabling the clinical picture to be placed in context and contribute to developing improved practice and thus better patient experiences.^13^

## Methods

Cases one and two were identified after contact with the Encephalitis Society (a global charity that raises awareness of encephalitis, participates and collaborates in research and provides support and information to professionals and those affected by the condition). Case 3 was identified after medical evacuation back to the UK, when the receiving hospital contacted the Walton Centre NHS Foundation Trust, Liverpool, UK. Cases two and three were both recruited into an NIHR Programme Grant on Encephalitis (the EncephUK study—approved by the National Research Ethics Service, East Midlands (11/EM/0442)) and followed the study protocol. Family members gave assent for recruitment. All the patients’ families independently approached the Encephalitis Society for help and support. All participants or their relatives gave written agreement to the publication of this report. Routine clinical data were collected, and face-to-face interviews were conducted with the patients and their family members during 2017–18.

## Results

### Patient One—Case Report

**Figure 1:**
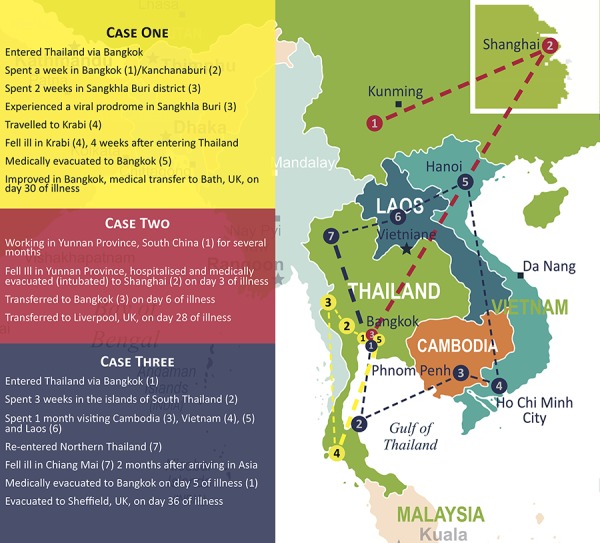
Map of the travel destination of the three cases, including medical evacuation routes within Asia

A 21-year-old female fell ill in Thailand in April 2014, 4 weeks into a trip planned to last several months. She had not been vaccinated against JE. She spent a few days in Bangkok, then 2–3 weeks in Kanchanaburi and Sangkhla Buri districts in Western Thailand where a febrile illness characterized by headache and myalgia developed (Figure 1). Accommodation was basic and she had many mosquito bites despite using diethyltoluamide (DEET) insect repellant daily. She partially recovered and then worsened a few days after arrival in Krabi. She was found unconscious by her mother in her hotel room and experienced a seizure on the way to hospital. At hospital, neurological examination revealed drooling and difficulty in breathing, but no other focal neurological signs. Shortly after arrival at hospital, she suffered a cardiac arrest. She was stabilized, the trachea intubated, and was transferred by air to Bangkok where lumbar puncture showed a raised opening pressure of 40 cm H_2_O and cerebrospinal fluid (CSF) pleocytosis of 40 leucocytes/mm^3^. Seizures persisted and were managed with phenytoin. C-reactive protein was 57 mg/L on admission and rose to 106 mg/L on Day 7 of illness. Other laboratory tests were unremarkable, and magnetic resonance imaging (MRI) of the brain was normal. Melioidosis serology was weakly positive prompting treatment with meropenem, though no abscesses were detected.

 She remained in hospital for 4 weeks. After extubation, stridor developed and necrotic tissue that was threatening the airway required removal by laser surgery. She underwent neuro-rehabilitation and made a good recovery. She was transferred by air to Royal United Hospitals Bath NHS Trust on Day 30 of illness, from where she was discharged the following day. JE serum IgG was positive, with negative IgM/IgG for dengue virus and West Nile virus, in the UK. Two and a half years after the illness onset, she experiences persistent fatigue and remains unable to see friends or go out in the evening; however, she has been able to complete a university degree and works full time as an assistant psychologist.

### Patient/Family Narrative

Interviews with the patient and her mother took place independently during 2017.

The patient did not remember much of the acute illness but recalled waking up in intensive care in Bangkok, where she describes it as ‘… all bright and the screens were all glass, and everyone was there’. She described ‘I couldn’t walk well, really confused … couldn’t really speak that well’. She could not recognize some visitors, including her brother. She recalled continuing to have seizures.

Following their return to the UK, the patient’s mother recalled her daughter sleeping a lot, experiencing bad headaches and memory difficulties. She reported her daughter ‘doesn’t think in the same way she used to’. Three years after becoming acutely unwell, and describing a ‘massive improvement’, the patient felt she was still in recovery and stated:

‘Life is different now … I have to plan my days ahead. It became apparent very quickly … how much my life had been affected by having the encephalitis. My doctors had informed me that returning to university might prove too difficult. I was unable to drive for a year due to the seizures. I had countless doctors’ appointments and I was unable to go out for long periods at a time. … I would feel exhausted after completing the smallest of tasks. I couldn’t remember a lot of my past, which obviously left me feeling very sad and confused. … My life has changed forever and I live with daily challenges that I once took for granted.’

Patient one sought travel health advice before the trip. Regarding JE vaccination, she recalled being told the risk was low and the provider had never heard of anyone affected by JE. She stated ‘… and because I’d never heard of it either I just thought I didn’t need to have it’. She wished she had more information, including written information, in particular about the severity of the condition, to better inform her decision.

### Patient Two—Case Report

A 31-year-old female from Wales developed a fever in July 2015, whilst spending several months working as an academic botanist in rural Yunnan Province, South China (Figure 1). She had received yellow fever vaccine in the past (details of the indication were not available) but not JE vaccine.

**Figure 2:**
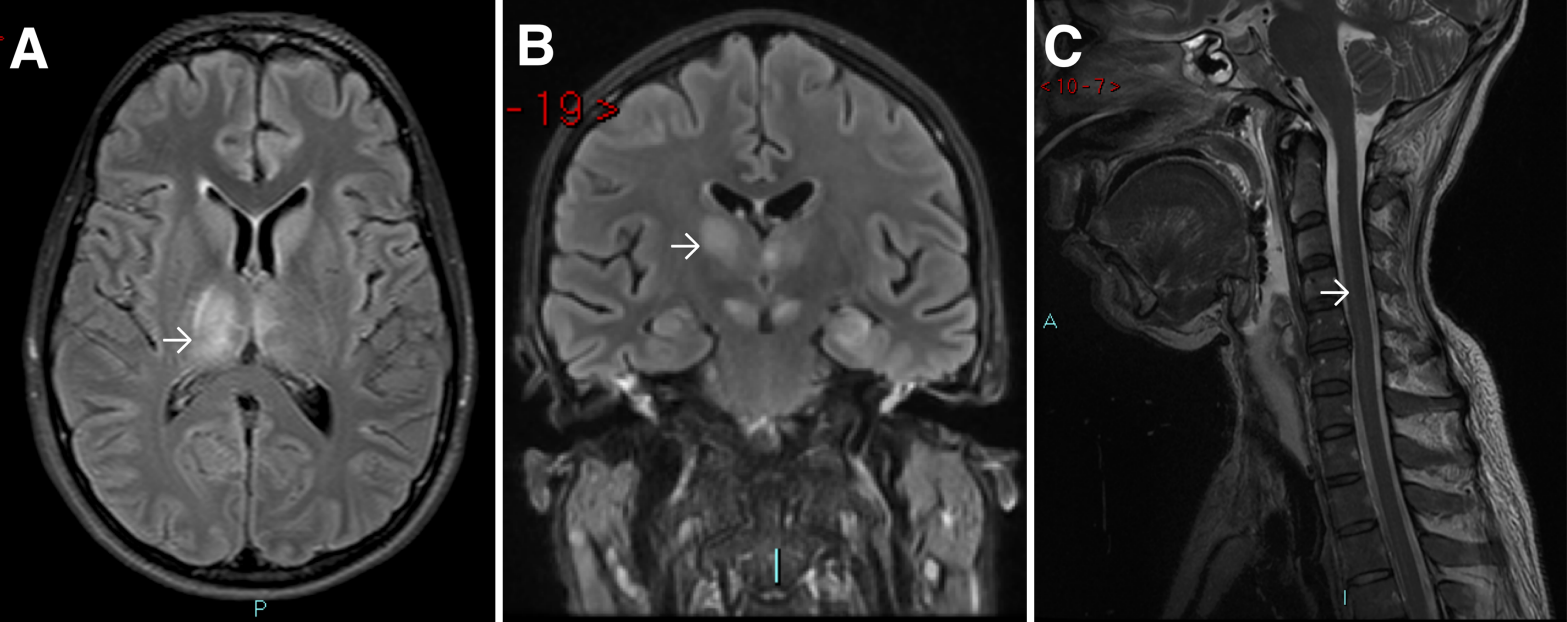
Neurological imaging of Patient two. (A), MRI scan performed in Bangkok on 18 July 2015 showing bilateral thalamic lesions, worse on the right (arrow). (B and C), MRI scan in Liverpool on 4 August 2015 showing persistent thalamic lesions (B, arrow) and high signal in the spinal cord (C, arrow)

The following day she was admitted to hospital after a collapse and seizure. She was intubated and transferred by air to Shanghai, and then to Bangkok on Day 6 of illness, where she was noted to be comatose with flaccid paralysis. CSF white cell count was 14 cells/mm^3^ and JEV IgM was detected in CSF. Her CT brain scan was normal; brain MRI on Day 14 showed high T2 signal in the thalami (Figure 2A). On Day 28 of illness, the patient was transferred to the Walton Centre NHS Foundation Trust, Liverpool, UK.

**Figure 3:**
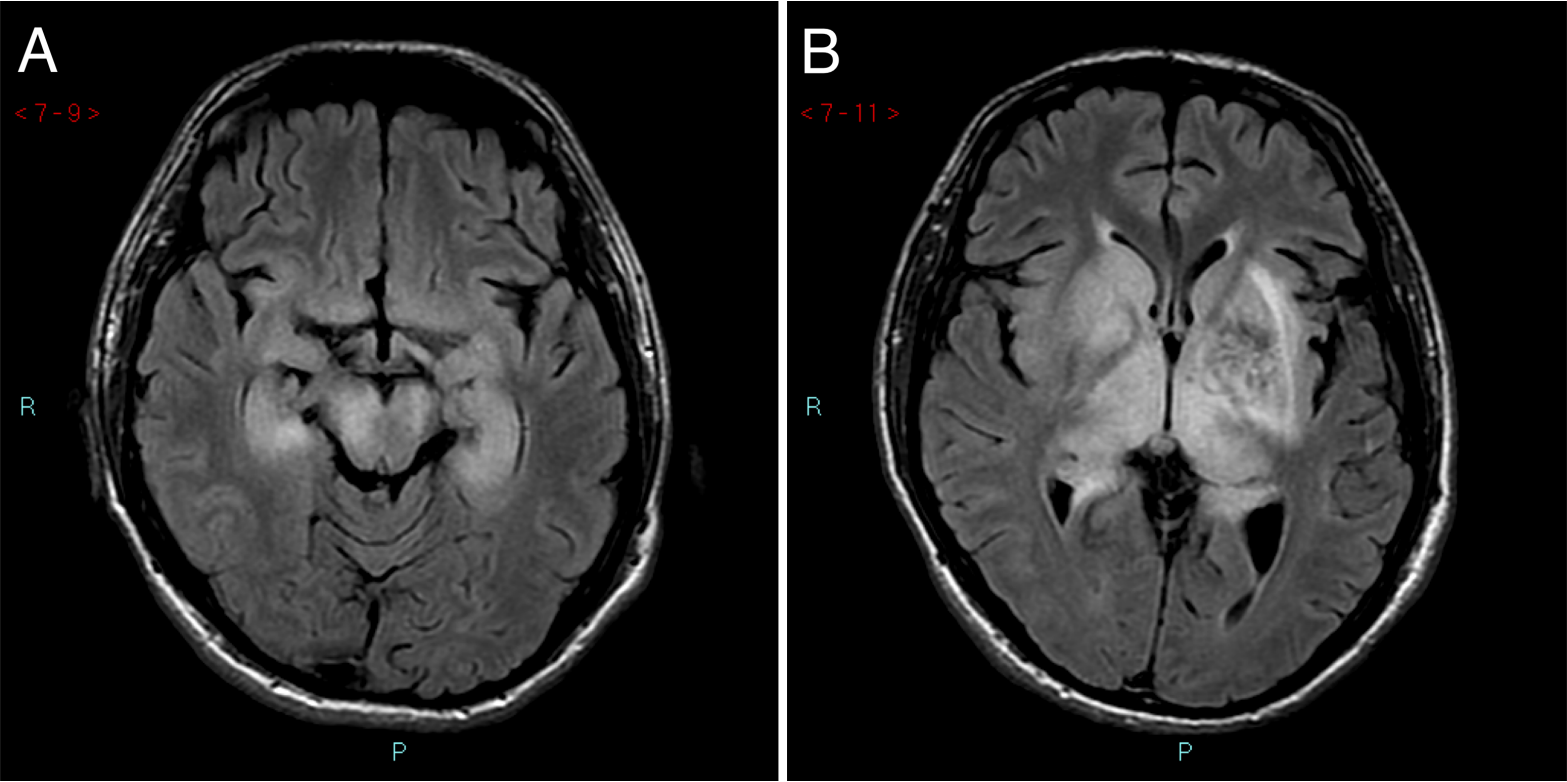
MRI of Patient three. (A), FLAIR images from an MRI scan performed in Bangkok on 5 August 2015 showing symmetrical high signal affecting the thalami and lentiform nuclei bilaterally. Evidence of radiosurgery to the left basal ganglia arteriovenous malformation with associated encephalomalacia within the left cerebral white matter and some haemosiderin deposition from the arteriovenous malformation is seen. (B), The abnormal area extends into the medial temporal lobes, the cerebral peduncles and into the brain stem

On arrival in Liverpool, she remained comatose. Neurological examination revealed reduced tone in all four limbs and areflexia. Lip smacking, abnormal mouth movements and dilated pupils were noted. Spontaneous ventilation was inadequate with respiratory rate five breaths per minute; therefore, invasive ventilation was continued. Withdrawal of anticonvulsants resulted in no change in her conscious level. A 24-hour EEG done after anticonvulsants were withdrawn showed only one brief period of seizure activity.

JEV IgG testing was strongly positive in serum and CSF. Thalamic lesions were still present on MRI (Figure 2B); high signal in the spinal cord suggestive of anterior horn involvement was also detected (Figure 2C). Given the gravity of her condition, and the poor outcome for severe JE, withdrawal of care was discussed, but the decision was taken to continue with active treatment.

She began to regain consciousness, and 2 weeks after transfer to the Walton Centre, she was able to follow simple commands. Upon re-examination at this point markedly reduced tone persisted, and power was Medical Research Council (MRC) grade 0/5 in all limbs. She remained ventilator dependent, with a tracheostomy, and unable to move her limbs or mouth. Her eyes would follow and she could close them on request, but she was effectively locked in.

She continued to improve slowly. At 2 years after the illness onset, she was fully conscious and able to talk in a whisper and take food orally, if spoon fed. Ventilation is still required. She had regained minimal movement (MRC grade 1/5 power) in the left arm and slightly more power (MRC grade 2/5) in the legs. She could not stand, or transfer, but could operate a physical therapy bike with her legs. She is currently living in a rehabilitation unit, unable to be left alone due to the ventilator, receiving nursing and rehabilitation, in addition to care from her partner.

### Patient/Family Narrative

A detailed interview was conducted with the patient’s father in 2017. A short interview was possible with the patient in 2018. The patient recalled little of her acute illness but perceived that she was unconscious with her eyes open, which she described as ‘strange’ and ‘scary’. She remembered her mother being there when she regained consciousness and how happy her mother was that she was able to recognize her.

Her father described feeling ‘absolutely devastated’ when he first saw his daughter, an image he said will ‘live with me forever more’. He described how the illness had ‘changed our lives forever … everything is just different … a lot of what we think about and do is centred around (her) because we want to help her in every way we can’.

The patient reports memory problems, fatigue and difficulties coming to terms with her disability. During the interview, she said ‘Have the (JE) vaccine, no matter what.’ Her father felt doctors need more knowledge, and that ‘People need to look it up and see what it entails and then ascertain whether you think it’s worth taking the risk (of not vaccinating), and if you could see (my daughter), you would know straight away that it is not.’

### Patient Three—Case Report

A 24-year-old male fell ill 4 weeks into an 8-week backpacking trip. Having visited Vietnam and Cambodia (Figure 1), he developed a febrile illness and was unable to lift his rucksack in northern Thailand in July 2015.

Past medical history was notable for a left thalamic haemorrhage secondary to an arteriovenous malformation in February 2014. This was treated with stereotactic radiosurgery, after which he made an excellent recovery with no significant neurological deficit or seizures.

One day after illness onset, he was admitted to hospital. Leukopenia and brain swelling on CT scan were noted. He deteriorated with seizures on the fifth day of illness and was intubated and transferred to Bangkok, where CSF analysis showed 30 mononuclear cells/mm^3^ and protein 124 mg/dl. An electroencephalogram was in keeping with non-convulsive status epilepticus. MRI on Day 12 showed bilateral high fluid-attenuated inversion recovery (FLAIR) signal in the deep grey matter (Figure 3), consistent with JE.^14^

The patient was transferred to the Royal Hallamshire Hospital, Sheffield, UK on Day 36. He remained unconscious and ventilator dependent through a tracheostomy. On examination, he was tolerating the tracheostomy tube, with eyes open spontaneously at times. Pupillary reaction and vestibulo-ocular reflexes were intact. Gaze was deviated to the left, with nystagmus on rightward gaze. There was no reaction to pain in the upper limbs, and reflex withdrawal from plantar stimulus both lower limbs. Limb tone was normal. After arrival, anti-JEV IgG in serum and total anti-JEV antibody in CSF were strongly positive. He regained eye opening and a flicker of movement in the left hand and was eventually moved to a rehabilitation unit where he remained ventilator dependent through a tracheostomy and was fed via gastrostomy. He is quadriplegic, with some slight movement in his hands and head. His family reports that he is able to smile, responds positively when friends visit and communicates by facial expressions and thumb movement only.

### Patient/Family Narrative

It was not possible to interview the patient, because of his condition, but his parents were interviewed separately during 2017.

The father described his son before the illness as ‘… really clever, a friendly lad with a lot of qualities people liked’. When they heard he was ill, both parents were shocked, with an overwhelming urge to get to their son as fast as possible.

They arrived in Bangkok 2 days after first learning he was ill. When his mother first saw her son in intensive care, she thought he was not going to live because it didn’t ‘look like he was in his body’. She took each minute of each day as it came, and it became clear he was going to live, but they had no sense of how he might be.

Now, his mother had given up her job to visit her son every day in his rehabilitation unit. She said:

‘The word devastating doesn’t cover it—it’s not anything you imagine, so I can’t put in to words how it is—it’s something you don’t expect to happen … the unexpected nature of it is what makes me have that shock first thing in morning and at the end of the day. Our aim is to keep him positive and moving ahead—you can’t look forward and you can’t look back—take each day as it comes.’

When asked about travel health, the patient’s mother said that this was not a risk they considered. Because her son had been to a travel clinic she assumed everything had been covered. His father said he ‘gets very angry that travel clinics don’t look in more detail in people’s travel plans and that they only point out likelihood [of disease] and not severity … this isn’t something that disappears—this is for life … another 5 minutes and the inoculation, and this wouldn’t be happening to us and other people—the way you are is changed forever’.

## Discussion

Although JE is an uncommon disease in travellers, these three cases illustrate the range of severity and outcomes, and the impact on survivors and their families. Between 1973 and 2008, there were 55 known cases of JE in travellers from non-endemic countries, of which at least 62% had an incomplete recovery.^15^ Since this report, a further 11 cases have been reported (reviewed by Caldwell *et al.*), of whom 4 died, 3 recovered and 4 were left with neurological sequelae.^16^ There have been no published cases in British travellers since 1992.^17^ The outcomes of the cases we describe were particularly poor in two cases, who remain ventilator dependent and incapable of independent living.

Case one was JEV IgG positive in serum but did not have other diagnostic testing performed. Therefore, the diagnostic evidence for JE is weaker than Cases two and three, although there was no other reasonable diagnosis, and JE remains the most likely diagnosis for this illness. There was also no abnormality on imaging, but this has also been reported at a similar stage of illness in a fatal case of JE.^18^ JE can be challenging to diagnose as virus or viral nucleic acid is usually not detected in readily available clinical specimens at the time of illness, and it is possible that JE is under diagnosed in returning travellers. For example, the Geosentinel survey has identified a number of cases of meningoencephalitis without a cause determined,^19^ including 48 cases returning from JE endemic areas between 2009 and 2018.^3^

The two cases with the worst outcome both had evidence of spinal cord involvement with JEV, exhibiting areflexic flaccid paralysis and ventilatory failure. Spinal cord involvement resulting in weakness has also been described in West Nile virus infection.^20^ Indeed, the ongoing need for mechanical ventilation in both our patients is reminiscent of severe poliomyelitis. However, both our patients have asymmetric residual deficits, similar to those originally described for JE in children in Asia.^21^ Our patients with spinal cord involvement also did not make a complete recovery, again similar to the majority of previously described patients. Unlike the previous report,^21^ both of our patients have had some degree of cognitive impairment, one with good recovery, the other less good.

Our interviews provide greater insight into the effects of JE. The most significant consequences of the illness are the lasting physical, psychological and emotional effects. Whilst the first patient made a good recovery, her life remained affected and was only manageable with forward planning. The other two patients remain severely impaired both physically and cognitively. Unfortunately, whilst some slight improvements may be anticipated, it is unlikely that they will recover significantly. Had these patients been residents of typical JE endemic areas in rural Asia, the illness would have likely been fatal.

All our cases were in adults. JE is mostly a disease of children in Asia, and there are few data comparing outcomes for children versus adults. Mild cases and asymptomatic infections were described in US service personnel stationed in Korea in the 1950s,^2^ as well as more severe cases.^22^ This work also suggested that adults from non-JE endemic areas were more susceptible to disease, following infection, than children native to these areas.^23^ However, small case numbers and the historical nature of these studies preclude any firm conclusion.

Most travellers to JE endemic regions do not get vaccinated; one study estimated that only 11% of eligible travellers received JE vaccine.^24^ The risk of developing JE in travellers is hard to determine. Asymptomatic infection is detectable but infrequent.^25^^,^^26^ Disease incidence will be even lower—estimated in European travellers at one case per 5.4 million visits to Asia.^27^ However, the risk will not be evenly distributed, a point emphasized by the finding of a risk of clinical disease (and not simply infection) of 3.7 per 1000 in a 3-month period among a population of US troops in Korea, in 1958.^2^ Studies of subclinical infection in Japan suggest an annual infection rate of 2.2%,^28^ but this infection rate could be as high as 5% in urban areas and 10% in rural areas in some provinces.^29^ Whether or not the actual risk of JE in travellers is changing is hard to determine. JEV transmission in urban and peri-urban settings has been reported,^4^^,^^30^^,^^31^ but basing travel vaccination recommendations on incidence data risks underestimating the possibility of disease due to an ‘epidemiologic silence’ created by successful vaccination programs.^32^ For example, JE vaccines were first introduced in China in the late 1960s, but only incorporated nationwide into the expanded program of immunization in 2008. Since then, JE incidence has decreased markedly,^33^ though environmental factors are if anything more conducive for the enzootic cycle of JEV.^16^

Any risk assessment for JE vaccination must, therefore, carefully consider the proposed itinerary and season of travel, as well as any predisposing conditions that might increase the risk of developing JE following infection with the virus, such as immunocompromise, extremes of age, pregnancy and conditions compromising the blood brain barrier, as well as the prospect of repeated visits.^3^^,^^34^ Our third patient’s arteriovenous malformation may have predisposed to JE through compromise to the blood–brain barrier. All three patients had clear indications for JE vaccination, according to several bodies that issue such guidance (WHO, CDC and PHE).^6–8^

All participants had strong views on the travel advice that had been given, stating they felt it had been insufficient or that whilst likelihood was explained to them, the severity of JE was not. Whilst there will be a significant component of hindsight bias influencing these views,^35^ they are completely understandable.

Although the risk of travel-acquired JE is low, the disease can be severe. An informed choice regarding vaccination must include an understanding of the potential consequences of acquiring JE, in addition to the likelihood, which will vary according to the exact itinerary and season of travel. The narratives provided in this paper demonstrate this is not always adequately done.

## Author’s contributions

LT collected and analysed clinical data and wrote the manuscript. AE performed interviews and co-wrote the manuscript. SD collected clinical data and supervised recruitment into EncephUK. ME collected clinical data and recruited Case 2 into EncephUK. BB, JH, AJ, PL and TS collected clinical data and provided clinical care for the cases. TS was chief investigator of EncephUK, analysed clinical data and co-wrote the manuscript. All authors read and commented upon the final draft of the manuscript, except BB.
